# Errata

**Published:** 1883-12

**Authors:** 


Errata.—November number, p. 355, line ninth from bottom, for *sepia*$^{2cm}$ read *sepia*$^{c}$. Page 320, line seventh, for *undilutable*, read *undilatable*. Page 318, line twenty-four, for *proven* read *provers*.

